# Supplementary material for: Splice-Junction-Based Mapping of Alternative Isoforms in the Human Proteome
Source: Cell Rep. Author manuscript; Available in PMC 2020 Jan 15. (PMC6961840; doi:10.1016/j.celrep.2019.11.026)

A

sp|O95425|SVIL_HUMAN|ENSG00000197321|MXE2|832|chr10|29531288|29532172|−1|r52|T1
RPELCTSHSETPTVDDEEKVDER q value: 5.2251e−05 Tr_novel:TRUE RefSeq_Novel:FALSE
Search result spec prec mz: 683.3151 Actual spec prec mz: 683.31512
Fragments matched per AA: 2.52 Proportion of top 20 peaks matched: 0.3

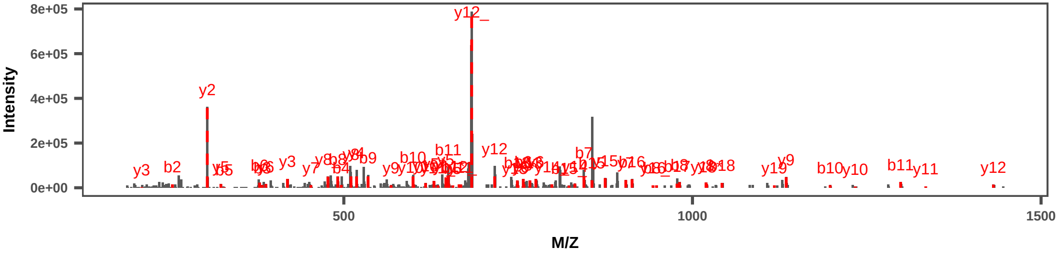

B

Scatterplot of predicted elution time
Fitting R2: 0.855
Novel peptide residual Z score: −0.415
Number of peptides: 1298

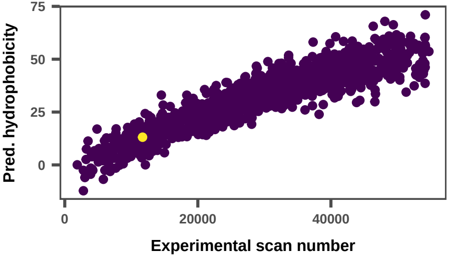

C

Distributions of residuals from best−fit line
of predicted RT vs Expt. scan number
Line: Z score of novel peptide
Z: −0.415

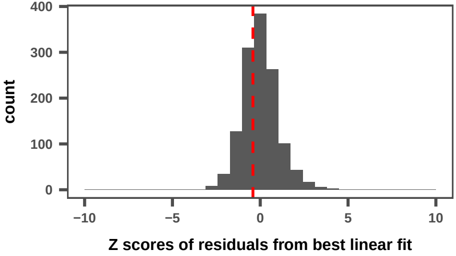

Supplement: 2 [file NIHMS1546469-supplement-2.zip › DF1/PXD010154/Colon/Colon_1_SVIL_RPELCTSHSETPTVDDEEKVDER.pdf]
